# Editorial: Emotional Disturbance and Brain Imaging in Neuropsychiatric Disorders

**DOI:** 10.3389/fpsyt.2021.632244

**Published:** 2021-03-17

**Authors:** Roberto Esposito, Fengyu Zhang, Maorong Hu, Ping Li, Wenbin Guo

**Affiliations:** ^1^Titano Diagnostic Clinic, Falciano, San Marino; ^2^Area Vasta 1, Azienda Sanitaria Unica Regionale (ASUR) Marche, Pesaro, Italy; ^3^Global Clinical and Translational Research Institute, Bethesda, MD, United States; ^4^Beijing Huilongguan Hospital and Peking University Huilongguan Clinical Medical Institute, Beijing, China; ^5^Department of Psychiatry, The First Affiliated Hospital of Nanchang University, Nanchang, China; ^6^Department of Psychiatry, Qiqihar Medical University, Qiqihar, China; ^7^Department of Psychiatry, National Clinical Research Center for Mental Disorders, The Second Xiangya Hospital of Central South University, Changsha, China; ^8^Department of Psychiatry, The Third People's Hospital of Foshan, Foshan, China

**Keywords:** structural MRI, functional MRI, depression, anxiety, phobia

## Introduction

Emotional disturbances, such as depression, anxiety, and phobias, are prevalent in patients with neuropsychiatric disorders, including somatization disorder (SD), major depressive disorder (MDD), general anxiety disorder (GAD), panic disorder (PD), schizophrenia, blepharospasm, and cervical dystonia (1–5). However, the neuropathology underlying the emotional symptoms in neuropsychiatric disorders remains unclear. Brain imaging techniques provide some unprecedented opportunities to study the neural mechanisms of emotional problems and neuropsychiatric disorders (6–10). This Research Topic convened 16 research articles based on the current understanding of emotional symptoms to explore the underlying neuropathological mechanisms and address important conceptual and methodological questions.

## Major Depressive Disorder

### Adolescent

Wu F. et al. explored functional and structural connectivity abnormalities within the amygdala-prefrontal circuit in first-episode medication-naive adolescents with MDD through resting-state functional magnetic resonance imaging (rs-fMRI) and diffusion tensor imaging (DTI). Rs-fMRI and DTI imaging data were acquired from 36 patients and 37 age-and sex-matched healthy controls (HCs). The patients showed decreased connectivity between the left amygdala and ventral prefrontal cortex (PFC) and lower fractional anisotropy (FA) in the left uncinate fasciculus. These findings suggest that both functional and structural abnormalities of the amygdala-prefrontal circuit may be involved in the neuropathophysiology of adolescent MDD.

Zhang et al. investigated how emotional context modulates the temporal dynamics of reward anticipation and feedback in adolescents. Electroencephalography (EEG) data from 35 patients with MDD and 37 healthy adolescents were recorded when performing a gambling task after being presented with emotional pictures. The study suggests that adolescents with MDD exhibited dissociable deficits in reward anticipation and gain or loss feedback that were distinctly modulated by emotional contexts, which might deepen our understanding of the modulation of emotional contexts on the dynamic temporal reorganization of the reward circuit in adolescents with MDD.

### Adult

Tao et al. investigated the subgenual anterior cingulate cortex (sACC) in patients with depression. Indeed, abnormal activity of the sACC is implicated as a potentially effective target for therapeutic modulation in treatment-resistant depression (TRD). The authors hypothesized that PFC areas with direct fiber connections to the sACC might be sites for effective treatment using transcranial magnetic stimulation (TMS). Two neuroimaging data sets were used to construct anatomic and functional connectivity maps using sACC as the seed region. One data set included magnetic resonance images from 20 HCs, and the other included MR images from 15 TRD patients and 15 additional HCs. Both left and right prefrontal cortex (PFC) is functionally connected to two regions relevant to depression, the sACC and the posterior cingulate cortex (PCC). These bilateral PFC sites may be targets for effective TMS treatment in TRD.

Chen et al. examined the changes in characteristics of affective network connectivity in patients with MDD before and after repetitive TMS treatment over the left dorsolateral PFC to assess how these connectivity changes are linked to the patient's clinical characteristics. rTMS can enhance affective network connectivity in patients with MDD, which is linked to emotional improvement. This study further suggests that the insula might be a potential target region for evaluating clinical efficacy for MDD in designing rational strategies for therapeutic trials.

Liu et al. examined whether the CACNA1C gene rs11832738 polymorphism and MDD had a potential interaction on untreated regional ALFF, and further determined whether the regional ALFF mediated the genetic association with MDD. With 116 patients and 66 HCs, they provided initial evidence for CACNA1C genotype-related alterations in brain function among patients with MDD, which could help better understand the neurobiological mechanisms underlying MDD. The study confirmed that the association of calcium channel dysfunction with MDD might be altered in functional brain activity.

### Late-Life Depression

Li et al. aimed to identify the voxel-based whole-brain functional connectivity changes in patients with late-life depression (LLD). With 50 patients and 33 HCs, the study indicated that the intrinsic abnormality of network centrality exists in a wide range of brain areas in patients with LLD. Late-onset depression patients differ from early-onset depression in cortical network centrality.

Krause-Sorio et al. conducted a pilot study of 22 older adults with depression who randomly received escitalopram/memantine or escitalopram/placebo treatment and tested with diffusion-weighted imaging (DWI) to investigate brain white matter integrity in the fronto-limbic-striatal tracts based on fractional anisotropy and treatment response. In bilateral anterior and posterior internal capsule tracts and bilateral inferior and right superior fronto-occipital fasciculus, higher fractional anisotropy was associated with more considerable improvements in depressive symptoms for escitalopram/memantine but not escitalopram/placebo. While the findings seemed promising, it has to be noted that the study was based on a small sample with some significant losses to follow-up. Therefore, further studies with adequate statistical power are needed in the future.

Wu Y. et al. aimed to examine altruism in patients with LLD and its neurobiological mechanism. Depressive patients seemed to show less altruistic behavior, while depression and age are two primary influencing factors of altruism. Kynurenine and its metabolites can cross the brain-blood barriers, impact the central nervous system, and play a significant role in psychiatric disorders, including depression. They investigated whether metabolites in the kynurenine pathway and white matter network topological features would influence altruistic behavior in patients with LLD. With 34 patients and 36 HCs, the study showed that patients exhibited a higher level of altruism and white matter global network properties than the HC. Kynurenic acid to kynurenine ratio was associated with the Dictator Game paradigm performance and network density in the patients. Kynurenine metabolism might play an important role in altruistic behavior in LLD.

## Bipolar Depression

Lu et al. performed a study of 27 HCs, and 36 patients with bipolar depression (BPD) treated with quetiapine. At baseline, the altered composition of gut microbiota and low B/E ratio was founded in patients with BPD, and Enterobacter spp count was significantly correlated to CD3^+^ T cells. Antipsychotic treatment significantly improved depressive symptoms and the balance of B/E. This finding suggests that the treatment effect on depressive symptoms might be partly through the improved gut microbiota balance.

Ren et al. investigated possible age-associated alterations of white matter integrity in adolescents and young adults with BPD aged 13–30. The findings provide neuroimaging evidence supporting a back-to-front spatiotemporal directionality of the altered development of white matter integrity associated with age in patients with BPD during adolescence/young adulthood.

## Comparison Between MDD and BD

Takahashi et al. explored abnormal melatonin secretion in patients with MDD and BPD. They employed MRI to examine pineal gland volumes and pineal cyst prevalence in 56 patients with MDD (29 currently depressed and 27 remitted patients), 26 patients with BPD, and matched HCs (33 for MDD and 24 for BD). The pineal gland was significantly smaller in the current MDD of non-melancholic depression than in the melancholic MDD. Pineal volumes negatively correlated to the severity of loss of interest in the current MDD. The medication used and the number of affective episodes were not associated with pineal volumes in the MDD or BPD. While the findings do not suggest that pineal volumes reflect abnormal melatonin secretion in affective disorders, they point to the possibility that pineal abnormalities are associated with clinical subtypes of MDD and its symptomatology.

## Schizophrenia

Luo et al. performed a study of dynamic functional connectivity strength (dFCS) at a different frequency with rs-fMRI data from 96 patients with schizophrenia (SZ) and 121 HCs. They found that relative to HCs, patients with SZ tend to have decreased dFCS in the salience, auditory, sensorimotor, and visual networks but increased dFCS in the cerebellum, basal ganglia, and prefrontal network consistently at low-frequency bands, which significantly interacted with disease status. However, no significant difference in dFCS was found in higher frequency. The study may provide potential implications for examining the neuropathological mechanism of SZ.

Duan and Zhu aimed to examine SZ research utilizing MRI through a bibliometric analysis of literature searched in PubMed from 2004 to 2018, divided into three 5-year periods. It showed that the utilization of MRI in SZ research was relatively diverse, but the theme clusters derived from each period reflect the evolvement from (1) the brain structure and its link to functional abnormality, metabolism, and antipsychotic efficacy and pathology; (2) the physiopathology mechanism and etiology of cognitive disorders including brain structure and function, default network, and psychology; and to (3) the neurobiology between SZ and other mental disorders, including the genetic and antipsychotic effect on brain structure and function. These findings provide useful information for developing future SZ research using MRI techniques.

## Substance Abuse

Yang et al. examined regional Cerebral Blood Flow (rCBF) alterations and their cognitive performance in unmedicated heroin-dependent individuals (HDIs). Voxel-wise whole-brain analysis of rCBF measured arterial spin labeling (ASL) perfusion MRI showed that relative to HCs. HDIs tend to have decreased rCBF in the bilateral cortical and subcortical inferior temporal gyrus, medial frontal gyrus (MFG), orbital medial frontal cortex, precuneus, posterior cerebellar lobe/declive, and right thalamus and posterior cerebella cortex, all of which were significant between HDIs and HCs in the ROIs analysis. The rCBF at MFG was significantly associated with cognitive performance measured by Trail Making Test. These findings suggest the MFG as a critical region in HDIs and suggest ASL-derived CBF as a potential marker for use in heroin addiction studies.

Sariah et al. performed an interesting study of whether chewing betel quid had acute and longer-term addictive effects in naive and dependent users. The rs-fMRI was performed in 24 male betel quid-dependent chewers and 28 male HCs before and promptly after betel quid chewing. They found that individuals who chronically used betel quid have higher functional connectivity in the frontal, parietal, and temporal brain regions relative to HCs. However, the acute effect was observed in naive Betel-quid chewers with increased functional connectivity in some visual cortical areas of the superior and right middle occipital gyrus and subcortical caudate, putamen, pallidum, and thalamus.

## Other Psychiatric Disorders

Seok and Cheong aimed to identify gray matter deficits and functional alterations using voxel-based morphometry and fMRI analyses of 15 men with intermittent explosive disorder (IED) and 15 age sex-matched HCs. Gray matter volume and brain activation while viewing the anger-inducing films were measured using 7T MRI. Men with IED had significantly reduced gray matter volume in the insula, amygdala, and orbitofrontal area relative to HC; gray matter volume in the left insula was negatively correlated with composite aggression scores. fMRI showed that men with IED showed greater activation in the insula, putamen, anterior cingulate cortex, and amygdala during anger processing. Left insula activity was positively correlated with composite aggression scores. These findings collectively suggest that structural and functional alterations in the left insula are linked to IED, thereby providing insight into IED's neural mechanisms.

## Conclusions and Future Perspectives

Emotional disturbance is mostly related to mood and anxiety disorders that are highly prevalent neuropsychiatric disorders and common in older adults of general populations (11). Neuroimaging is a critical tool for understanding the neural mechanisms and defining brain circuits underlying neuropsychiatric disorders. While most articles published in this specific theme employed observations of cases and healthy controls, two studies by Chen et al. and Krause-Sorio et al. examined neuroimaging in response to antidepressant treatment, which may provide a proof of concept that neuroimaging can predict pharmacological treatment response.

The advances in neuroimaging techniques and biotechnology have provided an unprecedented opportunity to study precision neuropsychiatric disorders in past years. Advanced neuroimaging techniques have helped to investigate brain structural and functional changes in neuropsychiatric diseases through MRI [fMRI, DTI, Perfusion Weighted Imaging (PWI), Cortical Thickness, Voxel-Based Morphometry], [Positron Emission Tomography (PET), and Magnetoencephalography (MEG)]; fMRI has in particular provided information about neural connectivity at rest (rs-fMRI) and during a task. Rs-fMRI represents a promising method for investigating brain *in-vivo* in physiological and pathological conditions due to both its spatial resolution compared to other methods, such as MEG and EEG, as well as the possibility of using it with poorly compliant patients due to their clinical condition. This allows us to highlight abnormalities at multiple levels across neural systems. Rs-fMRI studies have underlined that neuropsychiatric diseases show altered interactions between different RSNs, and disease progression and treatments may modify these interactions.

Even if neuroimaging techniques (especially rs-fMRI) represent valid tools to investigate neuropsychiatric diseases, results are often inconsistent, showing significant variability, which may present problems for identifying specific biomarkers of pathology. The lack of confirmed biomarkers might be due to different variables. These may include the heterogenicity of the neuropsychiatric disease, the diversities of data analysis technique used, and the limits intrinsic to rs-fMRI, such as the scarce possibility of monitoring patients' real mental content during the acquisition phase. Future studies should include standardized protocols for enrollment of sample size, reducing the heterogeneity observed. Integration of temporal and spatial imaging as well as clinical and molecular data may improve the identification of brain circuits related to neuropsychiatric disorders and treatment response.

Moreover, a combination of multimodal imaging tools, such as PET, structural MRI, and fMRI, can help understand brain circuits' structure and function. A PET scan can reveal metabolic and functional problems at a cellular level that MRI and fMRI cannot provide, which may be critical for some neuropsychiatric diseases. These precautions could provide more power in identifying disorder-specific neural alteration and help develop specific therapeutic interventions in future precision psychiatry.

## Author Contributions

All authors listed have made substantial and intellectual contributions to the topic and approved this editorial for publication.

## Conflict of Interest

The authors declare that the research was conducted in the absence of any commercial or financial relationships that could be construed as a potential conflict of interest.
